# Chemical Leaching from Tire Wear Particles with Various Treadwear Ratings

**DOI:** 10.3390/ijerph19106006

**Published:** 2022-05-15

**Authors:** Yoonah Jeong, Seokhwan Lee, Sang-Hee Woo

**Affiliations:** 1 Department of Environmental Research, Korea Institute of Civil Engineering and Building Technology (KICT), Goyang-daero 283, Goyang-si 10223, Korea; 2 Department of Mobility Power Research, Korea Institute of Machinery and Materials, Daejeon 34103, Korea; shlee@kimm.re.kr (S.L.); wsh@kimm.re.kr (S.-H.W.)

**Keywords:** tire wear particle, chemical leaching, treadwear rating, benzothiazole

## Abstract

Physical friction between a tire and the road surface generates tire wear particles (TWPs), which are a source of microplastics and particulate matter. This study investigated the trends of chemical leaching from TWPs depending on the treadwear rating of the tire. A road simulator was used to produce TWPs from tires with various treadwear ratings. Liquid chromatography–tandem mass spectrometry was used to analyze the chemical leaching from TWPs, with a particular focus on benzothiazole and its derivative 2-hydroxy benzothiazole. However, chemical mapping via high-resolution tandem mass spectrometry detected another derivative: 2-mercaptobenzothiazole. The benzothiazole groups were observed to have different leaching tendencies, implying that using benzothiazole as a marker compound may lead to incorrect TWP quantitation. The results of this research also suggest that the ecotoxicological influence of TWPs can vary with the treadwear rating of a tire.

## 1. Introduction

Tire wear particles (TWPs) are generated by mechanical friction between the road surface and a tire, and they can exist in two forms: non-airborne and airborne. A tire loses about 1–1.5 kg of its mass during its lifespan, and most TWPs (90.0–99.9%) are deposited on the road as non-airborne particulates. Only a small fraction of TWPs (0.1–10.0%) is emitted as airborne particulates [1]. Non-airborne TWPs are finally released into the environment by runoff or wastewater treatment effluent while changing in morphology and chemical composition due to aging and weathering. Meanwhile, airborne TWPs can be transported on the road, soil, or surface water by dry and wet deposition. The occurrence of TWPs has been confirmed in all environmental compartments [2]. Furthermore, the risk of TWPs to organisms has been assessed in terms of teratogenic, mutagenic, and estrogenic activities [3,4,5].

TWPs have drawn attention as a source of particulate matter (PM) and microplastics. Microplastics are defined as thermoplastic materials with a size of less than 5 mm [6]. Microplastics have been a significant social and environmental concern because of their vast consumption, ubiquity in the environment, and toxic effects [7]. Siegfried et al. (2017) and Rødland et al. (2022) identified TWPs as the most abundant source of microplastics exported into the sea via European water bodies (42%) [8,9], while Boucher and Friot (2017) proposed that synthetic fiber from laundry (35%) is a more significant source than TWPs (28%) [10].

Albeit a relatively small amount, airborne TWPs have become a popular research topic because of their role in atmospheric pollution. Technological advances have decreased the amount of PM from the exhaust gas, which has increased the relative importance of TWPs from non-exhaust gas. PM can be divided according to particle size: PM10 has a diameter of less than 10 µm, whereas PM2.5 has a diameter of less than 2.5 µm. There is some debate over the actual contribution of TWPs to PM. Tappe and Null (2002) reported that 5–7% of PM10 originates from TWP [11]. In contrast, Kreider et al. (2010) found that TWP makes a negligible contribution to PM because most TWPs have a diameter greater than 10 µm [12].

As a critical source of airborne PM and microplastics, the influence of TWPs on the environment needs to be fully investigated. A tire consists of rubbers such as styrene-butadiene rubber, butadiene rubber, and natural rubber; vulcanization agents including sulfur (S), zinc (Zn), polyaromatic hydrocarbons (PAHs), and thiazoles; filler materials including carbon black and silica; and protective agents including halogenated cyanoalkanes [1]. As tires contain a considerable amount of heavy metals, TWPs have been recognized as a major source of the metals being introduced into the environment [13,14]. However, qualitative and quantitative evaluations of TWP in the environment are still a challenge. The amount of TWPs released and their physicochemical properties depend on factors such as the road status (e.g., material, texture, and wetness), tire properties (e.g., composition, tire pressure, and accumulated mileage), and intensity of friction (e.g., speed, acceleration, and weight of vehicle). Even under the same conditions in a road simulator, tires from different manufacturers exhibited distinctive characteristics in terms of the average size, size distribution, and chemical composition of their TWPs [12].

According to the Uniform Tire Quality Grading (UTQG) system, the treadwear rating is an indicator of the tread wear, traction, and temperature of a tire. A higher treadwear rating indicates a longer lifespan for a tire. It can be assumed that tires with higher treadwear ratings release fewer TWPs. Previous studies have considered the influence of the treadwear rating on TWPs in terms of airborne PM [15,16]. Their results indicated that a lower treadwear rating leads to a higher mass loss and generation of PM10 but not to a statistically significant extent. Even under the same conditions in a tire wear simulator, TWPs generated from different brands exhibited different patterns of material loss and PM emission. The roughness of the road surface can enhance the wear rate by two-to-three orders of magnitude [17,18].

This study focused on the effect of the treadwear rating on the chemical leaching from TWPs. TWPs were generated from a tire wear simulator by using tires with various treadwear ratings. Experiments were performed in a clean chamber to eliminate other airborne particulates. Both targeted and non-targeted analyses were used to evaluate the chemical leaching. Benzothiazole (BT) and 2-hydroxy benzothiazole (2-OH-BT) were selected for targeted analysis because rubber products contain BT and its derivatives to enhance abrasion resistance and accelerate vulcanization [19]. Previous studies have confirmed that TWPs leach BT [20]. A lower treadwear rating can be assumed to lead to TWPs with a larger amount of BT, but this has not been investigated in a controlled experimental setup. The results of this study can help clarify the ecotoxicological effects of TWPs depending on the treadwear rating and the viability of BT as a marker compound for TWP quantitation.

## 2. Materials and Methods

### 2.1. Tire and Target Compounds

Commercial tires (Kumho, Korea) were used with the same UTQG specifications for the traction and temperature. The only differences were in the treadwear rating: 250, 350, 500, and 700. The tire pressure was maintained at 36 psi.

Based on the results of a previous study, a quantitative analysis was performed on two compounds: BT and 2-OH-BT. For chemical analysis, high-purity BT and 2-OH-BT (>99.9%) were obtained from Sigma-Aldrich (St. Louis, MO, USA). Mass spectrometry (MS)-grade methanol, acetonitrile with 0.1% (*v*/*v*) formic acid, and water with 0.1% (*v*/*v*) formic acid were also purchased from Sigma-Aldrich.

### 2.2. Tire Wear Particle Generator

**Figure 1:**
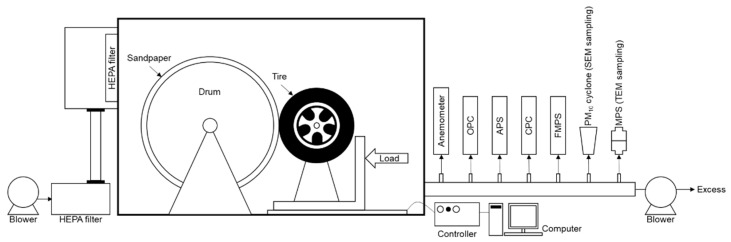
Schematic of the road simulator used for tire wear particle (TWP) generation.

Figure 1 shows the customized TWP generator used in this study, which comprised a rotating drum, control system, and test tire. Details on the TWP generator are provided elsewhere [21]. Briefly, the rotating drum (diameter: 1.2 m) was covered with 80 grit sandpaper to reproduce the conditions of asphalt pavement. The generator was placed in an enclosed chamber (length: 3.5 m, width: 2.4 m, height: 2.2 m) to exclude any extraneous material. The lateral load, drum speed, tire speed, and slip speed were controlled for TWP generation, and the driving speed was adjusted by using the rotating speed of the drum. Detailed characterization of the TWPs generated by the TWP generator is available elsewhere [21].

### 2.3. Chemical Analysis

The TWP morphology was monitored by scanning electron microscopy with energy-dispersive X-ray spectrometry (SEM–EDX) using JSM-7610F-Plus (JEOL Ltd., Akishima, Japan). For analysis of the TWP leachate, TWPs were extracted according to the method described by Zhang et al. (2018) [20]. In brief, the TWP mass was weighed accurately before extraction and was then placed in a headspace vial with screw caps. A mixture of methanol and high-performance liquid chromatography (HPLC)-grade water (5:3, *v*/*v*) was added to the headspace vial. Each headspace vial was ultrasonicated for 1 h, which was repeated three times. The supernatant was analyzed directly using liquid chromatography–tandem mass spectrometry (LC–MS/MS). A Thermo Vanquish LC system coupled with a TSQ Altis Triple quadrupole mass spectrometer (Thermo Fisher Scientific, Waltham, MA, USA) was used for the targeted and non-targeted analyses. A CORTECS C18 column (Waters Corp., Milford, CT, USA) (2.1 × 150, 1.6 µm) was employed for sample separation and was kept at a temperature of 45 °C. A binary mobile phase of A (0.1% formic acid in water) and B (0.1% formic acid in acetonitrile) was applied with the following gradient program: 10% B at 0–0.5 min, 100% B at 5.5–6.0 min, and 10% B at 6.5 min; stop time for 10 min; and post-time for 1 min. An aliquot of 3 µL was injected at a flow rate of 0.3 mL/min. MS was performed in the positive electrospray ionization (ESI) mode. For structural identification in the non-target analysis, MS was performed in full-scan mode between 50 and 2000 *m*/*z*. Prior to sample analysis, instruments were calibrated against calibration standards to ensure their accuracy and sensitivity. For multiple-reaction monitoring (MRM) of LC–MS/MS, each compound was confirmed based on MRM pair transitions from the precursor ion to the product ions as well as the retention time. The LC–MS/MS analysis was conducted for all treadwear ratings. The results are presented in Table 1.

## 3. Results

### 3.1. Scanning Electron Microscopy–Energy-Dispersive X-ray Spectrometry

**Figure 2:**
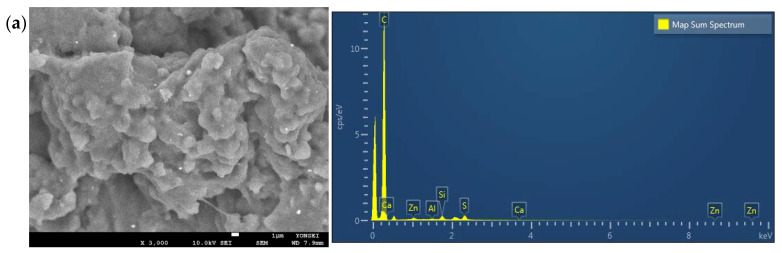

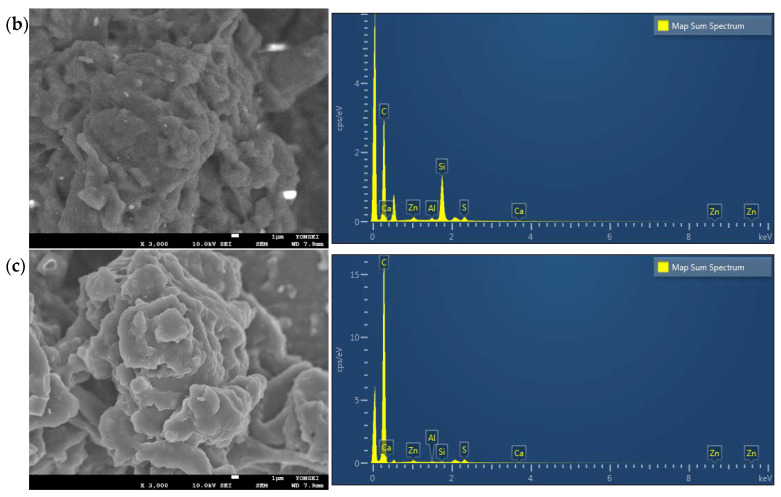
SEM images and related EDX spectra of TWPs generated with different treadwear ratings: (**a**) 250, (**b**) 350, and (**c**) 700.

SEM–EDX was used to characterize TWPs generated with different treadwear ratings: 250, 350, 500, and 700. The TWPs had diverse sizes and irregular shapes. Visible particles were larger than 5 µm in size, and tiny particles were smaller than 10–100 nm. Some small particles were also observed to aggregate by electrostatic interaction. Figure 2 shows that the TWPs were mainly coarse, irregular, jagged, and round in shape. Some of the TWPs had elongated shapes with sharp edges. In this study, the TWPs were generated in a road simulator in a laboratory, which led to a relatively consistent size and shape.

The EDX mapping results indicated that the TWPs contained a wide range of elements, including C, Ca, Al, Cl, Cu, Fe, Mg, Na, Ni, O, Si, Si, and Zn, where the major elements on the TWP surfaces were C, Si, S, and Zn. Generalizing the elemental composition of the TWPs based on the EDX mapping results is difficult because of the limited number of measurements. Still, the elemental compositions were similar for the various treadwear ratings. This result generally agrees with previous studies [12,15,22,23,24]. The proportion of C was over 85% for all samples because carbon black was one of the main components in the tires. Meanwhile, SiO_2_ was also employed as a reinforcing agent. The tires contained 0.5–2% vulcanization agents, activators, and accelerators, which included S, Zn, and Ca. Among these, Zn has been considered an indicator of TWP because of its high detection rate and high concentration. A minor difference between previous studies and this study is the TWP sampling method. Previous studies obtained TWP samples from actual roads, while TWP samples were generated in a clean chamber in this study. The EDX mapping results did not allow for quantitative profiling, but the low detection/depletion of Na, K, and Ca in the TWPs of this study probably be attributed to the difference in sampling conditions.

### 3.2. Quantitative Analysis of Chemical Leaching from TWP

**Figure 3:**
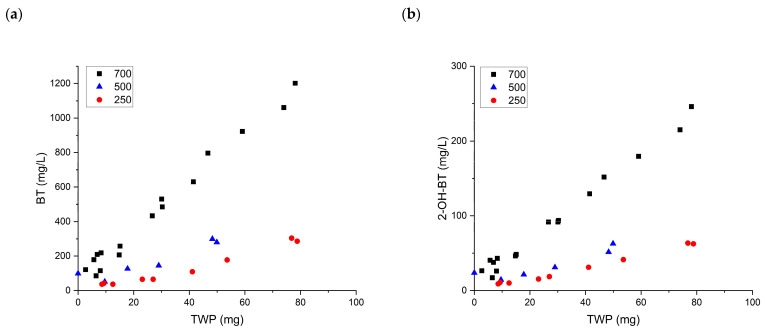
Concentrations of (**a**) BT and (**b**) 2-OH-BT in TWPs at various treadwear ratings.

A quantitative analysis of BT and its derivative 2-OH-BT was performed with the TWP leachate. Figure 3 shows that the BT and 2-OH-BT concentrations had linear relationships with the amount of TWP. The BT concentration was more significant than the 2-OH-BT concentration for all treadwear ratings. This difference in concentrations is reasonable because 2-OH-BT is generated at high temperatures, which can occur when there is strong friction between the road and tire. Although the specific contents of BT and 2-OH-BT in tires vary depending on the manufacturer, Zhang et al. (2018) confirmed that different brands of tires contain BT and BT derivatives, and their environmental matrix showed that BT had the highest concentration and highest detection rate. BT and 2-OH-BT contributed over 50% of the total mass fraction of BT groups. In general, the BT concentration was 2.5 times greater than 2-OH-BT, which made up the second-largest proportion among BT groups in the tire.

A high correlation coefficient (r2) would imply that BT leaching from TWP is sensitive to the amount of TWP generated. The number of samples varied between treadwear ratings, but the highest correlation coefficient was obtained for TWPs from tires with a treadwear rating of 700 (>0.98 for BT and 2-OH-BT). The slopes for the linear relationships differed according to the treadwear rating and target analyte. The steepest slope was obtained with a treadwear rating of 700 for both BT and 2-OH-BT. Concentration differences between treadwear ratings of 250 and 500 were minor for both compounds; BT had slopes of 3.78 and 4.59 for treadwear ratings of 250 and 500, respectively, in contrast to a slope of 14.37 for a treadwear rating of 700. The leaching of BT was over three times higher at higher treadwear ratings. There are several explanations for this. First, the tires with treadwear ratings of 250 and 500 contained similar amounts of BT, while the tire with a treadwear rating of 700 contained considerably more BT. Second, even if tires with different treadwear ratings had similar amounts of BT, the leaching tendencies of BT and 2-OH-BT could vary depending on the treadwear rating. The leaching rate of a compound is controlled by its solubility and diffusion gradient in a solution. BT and 2-OH-BT have similar solubility levels at a pH range of 4–6, but BT is more soluble at lower pH, and 2-OH-BT is more soluble at higher pH.

Tires with higher treadwear ratings have better wear performance. Accordingly, a lower treadwear rating induces higher tire tread loss and wear rate (Park et al., 2018; characteristics of the tire). Under rough driving conditions, coarse TWPs are generated in large quantities. A smaller amount of airborne PM is generated, but more particles are discharged on the road surface. It is reasonable to assume that chemical leaching is affected by the TWP size. More chemical leaching can be expected from fine particles because of the increased surface area relative to their volume. Once released TWPs settle, they undergo aging, degradation, and irregular physical friction, which eventually cause their further breakdown into smaller pieces.

The estimated TWP concentration varies depending on the marker compound used. Zhang et al. (2018) reported that PM originating from TWPs contains 22.0–26.6 µg/g of total BT [20]. Unice et al. (2013) estimated a broad range of TWP concentrations of 5800–11,600 µg/g when using SBR/BR and NR pyrolysis [25]. Meanwhile, Wik et al. (2008) estimated 9200–18,400 µg/g when using Zn [4]. Kumata et al. (1996) and Kumata et al. (2002) calculated a TWP concentration of 2800–7800 µg/g based on the leaching of 2-(4-morpholinyl)benzothiazole and N-cyclohexylbenzothiazol-2-amine [26,27]. In this study, the estimated TWP concentration had a range of 5346–19,432 µg/g. Such a wide gap between the estimated TWP concentration in this study and those in previous studies confirmed that the use of marker compounds for TWP quantitation is not a reliable and accurate approach.

**Figure 4:**
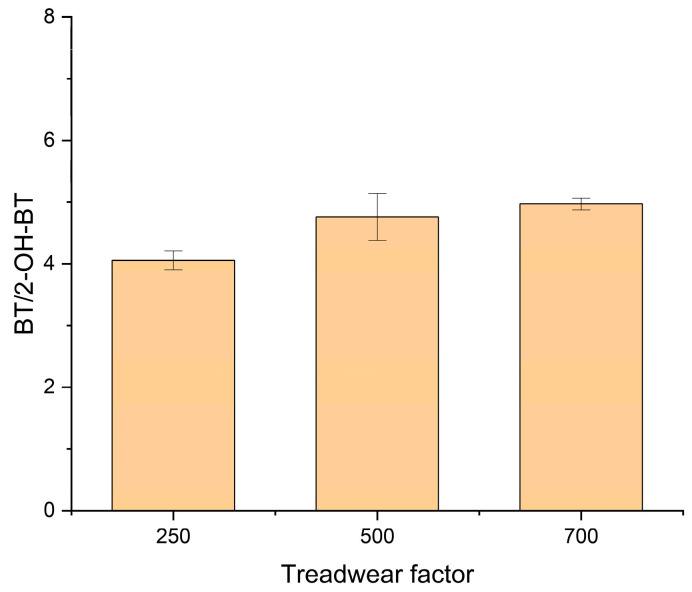
BT/2-OH-BT ratios with different treadwear ratings.

Figure 4 presents the ratio of BT to 2-OH-BT concentrations in this study. The ratio slightly increased with the treadwear rating, but the statistical analysis was not performed owing to the limited number of samples. 

### 3.3. Qualitative Analysis of Chemical Leaching from TWP

Non-targeted analysis was performed to observe the chemical spectrum of the TWP leachate. Interestingly, BT and 2-OH-BT were not detected, but 2-mercaptobenzothiazole (MBT) and benzothiazole-2-sulfonic acid (BSA) were detected in all TWP samples. Table 2 gives the results for these two compounds. As MBT is widely used in rubber production [28], it has been detected in atmospheric PM10 samples, road dust, and roadside soil [20,29]. It is worth noting that there has been limited research on TWP. Although it is difficult to generalize a particular compound as the most detectable and/or leachable among BT groups, Avagyan et al. (2014) found that the MBT concentration was 11 times higher than that of BT in tire debris. Among various derivatives of BT, BSA is one of the most dominant substances detected in municipal wastewater [30]. 

## 4. Implications of This Study

### 4.1. Environmental Risk of TWPs

Difficulties with generalizing TWPs in terms of physical characteristics such as shape and chemical properties have hampered their quantitation. The environmental risk assessment of contaminants considers both exposure and hazardousness [31]. Accordingly, both the amount of TWPs released into the environment and their toxicity need to be evaluated. Various pieces of equipment have been used for TWP quantitation, including pyrolysis gas chromatography–mass spectrometry (py-GC–MS), Fourier transform infrared spectroscopy (FTIR), Raman spectroscopy, single-particle analysis, and thermal extraction desorption GC–MS [3,32]. As the physical characteristics of TWPs vary, TWP quantitation methods are not consolidated but diversified. Each analytical technique has a different measurement index.

The toxicity of TWPs can be divided into two parts: that of TWP itself and that of the TWP leachate. Many studies have been performed on the adverse effects of microrubber and chemical leachate from TWPs [33,34]. As a microrubber, TWPs have shown acute toxicity in freshwater amphipods and earthworms [35,36]. Small planktonic crustaceans (e.g., *Daphnia magna*) and amphipod crustaceans (e.g., *Gammarus pulex*) may ingest TWPs [4,37]. The toxic influence of the chemical cocktail from TWP leachate has also been demonstrated [5,34,38]. The mortality of human alveolar lung cells and DNA damage was observed when cells were exposed to TWP extract for 72 h [5].

Among the various chemical leachates from TWPs, BT and heavy metals are commonly considered key drivers of toxicity. TWPs are among the major sources of BT and heavy metals introduced into the environment [13,14]. BT and its derivatives are recalcitrant to biodegradation in general, which has led to their frequent detection in the environmental matrix [39,40,41,42]. Several compounds of the BT group have been pointed out as persistent, mobile, and toxic (PMT) substances [43], including BT, MBT, 2-(morpholinothio)benzothiazole, and 2-(thiocyanomethylthio)benzothiazole. This indicates that these PMT substances may potentially experience long-range transport over long time scales once TWP is released into the environment. Due to the technically tricky removal of BT, Domínguez et al. (2012) measured similar BT concentrations in the influent and effluent of a wastewater treatment plant (WWTP) [39]. The distribution and transformation of BT and its derivatives in various environmental matrices are difficult to estimate. Wik et al. (2009) demonstrated an inconsistent and wide range of exposure concentrations from TWP leachate [4]. Herein, the occurrence of other derivatives of BT such as hydroxybenzothiazole (HBT), methylthiobenzothiazole (MTBT), aminobenzothiazole (ABT), and MBT has not been fully considered. For example, further degradation of ABT and OHBT is not expected because of their stable nature, compared with BT. In addition, the removal efficiency of each compound in a WWTP varies. From computational toxicity assessment, MBT and BSA exhibit a similar probability of thyroperoxidase inhibition, which is lower toxicity than other transformation products of MBT [44]. Therefore, obtaining reliable assessment results regarding the environmental risk of TWPs is difficult. The toxicity of TWP and TWP leachate can be biased when various tires (e.g., brand and treadwear ratings) have not been considered. The ecotoxicological influence of tires should be considered when scrap tires are reused as a construction material [45].

### 4.2. BT as a Marker Compound of TWP

There have been several attempts to analyze and establish the marker compound of TWPs. An ideal marker compound should satisfy several requirements. First, the marker compound should be in all tires regardless of the manufacturer. Second, the marker compound should be a distinct compound released from TWPs that does not have various sources. If the marker compound has multiple sources in the environment, it would be difficult to correlate the concentration of the marker compound with TWP emissions. Third, the marker compound should not degrade easily to maintain a consistent concentration and keep a straightforward analytical process.

BT and Zn have been considered the main candidates for TWP marker compounds. Both Zn and BT are present in TWP leachate in relatively high quantities. Kloeckner et al. (2019) employed Zn determination combined with density separation for TWP quantitation [32]. Other marker compounds considered in previous studies include S [46], cyclohexenylbenzene [3], vinylcyclohexene, and dipentene [25]. Previous studies have shown that BT could be used as an indicator of the presence of TWP. However, using the BT concentration for TWP quantitation is not appropriate because the BT concentration in TWP leachate depends on both the manufacturer as well as treadwear rating. In addition, using different marker compounds results in a broad range of estimated TWP concentrations. Although Wagner et al. (2018) estimated that the released amount of TWPs is 1.33 × 10^5^ tons/year, no specific data are yet available to quantitate the amount of TWPs in the environment and their influence [13]. An alternative approach to TWP quantitation could be tracking the changes in the tire thickness compared with the initial state.

## 5. Conclusions

Increasing traffic can be assumed to increase the amount of TWPs released into the environment, and the associated environmental risk is a matter of growing concern. TWPs are regarded as a new type of hazardous material in terms of microplastics and PM. In this study, a road simulator in a clean chamber was used to investigate the TWPs generated from tires with various treadwear ratings. Targeted and non-targeted analyses were performed to evaluate the effect of the treadwear rating on TWP leachate. Considerably higher concentrations of BT and 2-OH-BT were found in TWPs at a treadwear rating of 700 than at lower treadwear ratings of 250 and 500. The results of this study led to two main observations. First, environmental risk assessments of TWPs need to consider the amount of TWPs in addition to the leaching tendencies of chemicals in TWPs. Second, the application of BT or other chemicals in the BT group as a marker compound could lead to erroneous results regarding TWP quantitation. The results of this study indicate that the use of marker compounds for TWP quantitation is, in general, a questionable approach. This implies the need for an alternative approach to verifying the amount of TWP released and the environmental impact.

## Figures and Tables

**Table 1:** Parameters of BT and 2-OH-BT used for LC–MS/MS analysis.

Compound	Retention Time (min)	Retention Time Window (min)	Precursor Ion (*m*/*z*)	Product Ion (*m*/*z*)	Collision Energy (V)	Radio Frequency Lens (V)
BT	4.90	2	136	65.000	33.56	66
				77.054	26.61	66
				109.113	25.81	66
2-OH-BT	4.63	2	152	80.125	28.17	72
				92.054	20.04	72
				124.113	20.08	72

**Table 2:** Analytical parameters of MBT and BSA.

Chemical	CAS Number	Chemical Structure	Formula	Retention Time	*m*/*z*
Benzothiazole-2-sulfonic acid	941-57-1	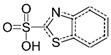	C_7_H_5_NO_3_S_2_	4.78	215.0
2-mercaptobenzothiazole	149-30-4	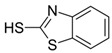	C_7_H_5_NS_2_	8.33	167.0
